# The Coastal Seafloor Microbiota Is Structured by Local Selection of Cosmopolitan Taxa

**DOI:** 10.1111/1758-2229.70123

**Published:** 2025-06-19

**Authors:** Knut Rudi, Tonje Nilsen, Ragnhild Pettersen, Nigel Brian Keeley, Jessica Louise Ray, Sanna Majaneva, Morten Stokkan, Anja Hervik, Inga Leena Angell, Melcy Philip, Julie Martin, Maud Ødegaard Sundt, Lars Gustav Snipen

**Affiliations:** ^1^ Norwegian University of Life Sciences Ås Norway; ^2^ Akvaplan‐Niva Tromsø Norway; ^3^ Institute of Marine Research Tromsø Norway; ^4^ Aqua Kompetanse AS Norway; ^5^ Stim as Lofoten Norway

## Abstract

Understanding the assembly processes of the coastal seafloor microbiota is crucial for gaining insights into how ocean ecosystems work. In our study, we addressed the question about how local selection affects the global distribution of coastal seafloor microorganisms. We identified two main clusters of samples by examining the geographical distribution of 356 high‐quality prokaryote metagenome‐assembled genomes (MAGs) from 94 coastal samples collected along the Norwegian and Icelandic coasts. There was no identifiable correlation between the abundance of MAGs and the geographic distance between them central to the identified clusters (no distance decay). In contrast, noncentral MAGs demonstrate a pronounced distance decay. We also observed significant functional differences between the two sample clusters. One cluster showed enrichment in functions such as dissimilatory nitrate reduction to ammonium (DNRA), acetoclastic methanogenesis, thiosulphate conversion and acetate and butyrate metabolism. The other cluster was enriched in propionate metabolism, nitrite oxidation to nitrate and cobalamin‐dependent carbon fixation. These results suggest that localised environmental selection acts on cosmopolitan taxa to shape seafloor microbiota. Our findings therefore profoundly impact the understanding of seafloor ecological processes and their management.

## Graphical abstract

Illustration of how microbial networks are selected along the Norwegian coast, with only one network type being present at Iceland.

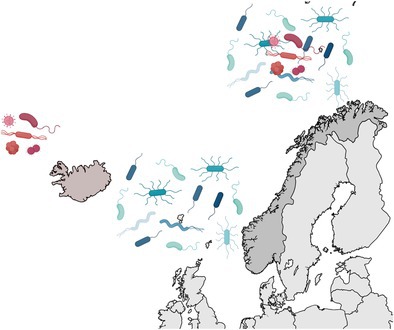

## 1 Introduction

The coastal seafloor represents one of the richest and most important marine ecosystems on Earth (Snelgrove 1999). The high biodiversity and productivity of coastal regions can be attributed to the shallow seafloor in combination with the input of essential nutrients from land, where seafloor microbes are key in nutrient remineralisation for supporting macroscopic life (Terhaar et al. 2021). Despite being under severe pressure from climate change and anthropogenic pollution (Lam‐Gordillo et al. 2020), we have very limited knowledge about the mechanisms underlying the assembly and function of the coastal seafloor microbiota (Nemergut et al. 2013; Orsi 2018).

Both anthropogenic and geothermal activity can have major impacts on the microbiota at the seafloor (Keeley et al. 2019; Laiolo et al. 2024; Orsi 2018; Pettersen et al. 2022; Zhou and Ning 2017). Geothermal activity contributes to the accumulation of reduced sulphur compounds in the environment (Dick 2019), while anthropogenic activity is a source of both organic carbon and sulphides (Labbate et al. 2016). In the North Atlantic region, the Mid‐Atlantic Ridge is a site of high geothermal activity (Quon and Ehlers 1963), with Iceland being situated in the middle (Denk et al. 2011). The Norwegian coastline, in contrast, is recognised as a coastal region impacted by intensive aquaculture activity rather than geothermal activity (Olaussen 2018).

Recent evidence suggests that the coastal seafloor redox chemistry and microbial diversity show bimodal distributions, with major differences even in closely spaced sites, with sulphides and organic carbon suggested as the localised drivers of seafloor microbial assemblage composition (Nilsen et al. 2024; Pettersen et al. 2022; van de Velde et al. 2020). The seafloor is exposed to microbial dispersal through global and local ocean currents (Girguis 2016). For coastal ecosystems, we do not know the extent to which global dispersal affects local microbial assemblies, and in particular dispersion between coastal regions and deep protected fjords, such as those along the Norwegian coast (Meyer et al. 2020).

The primary aim of the current study was to investigate microbial community assembly mechanisms in coastal regions separated by large geographical distances. This was done by analysing the distribution of 356 previously identified MAGs (Nilsen et al. 2025) from 94 sampling sites along the Norwegian and Icelandic coast. We addressed whether the microbial community composition and function were primarily structured by the selection of cosmopolitan taxa (widely distributed but locally selected) (Becking 1934), or whether the community composition and function were predominantly determined by local strains (Volkov et al. 2003).

**FIGURE 1:**
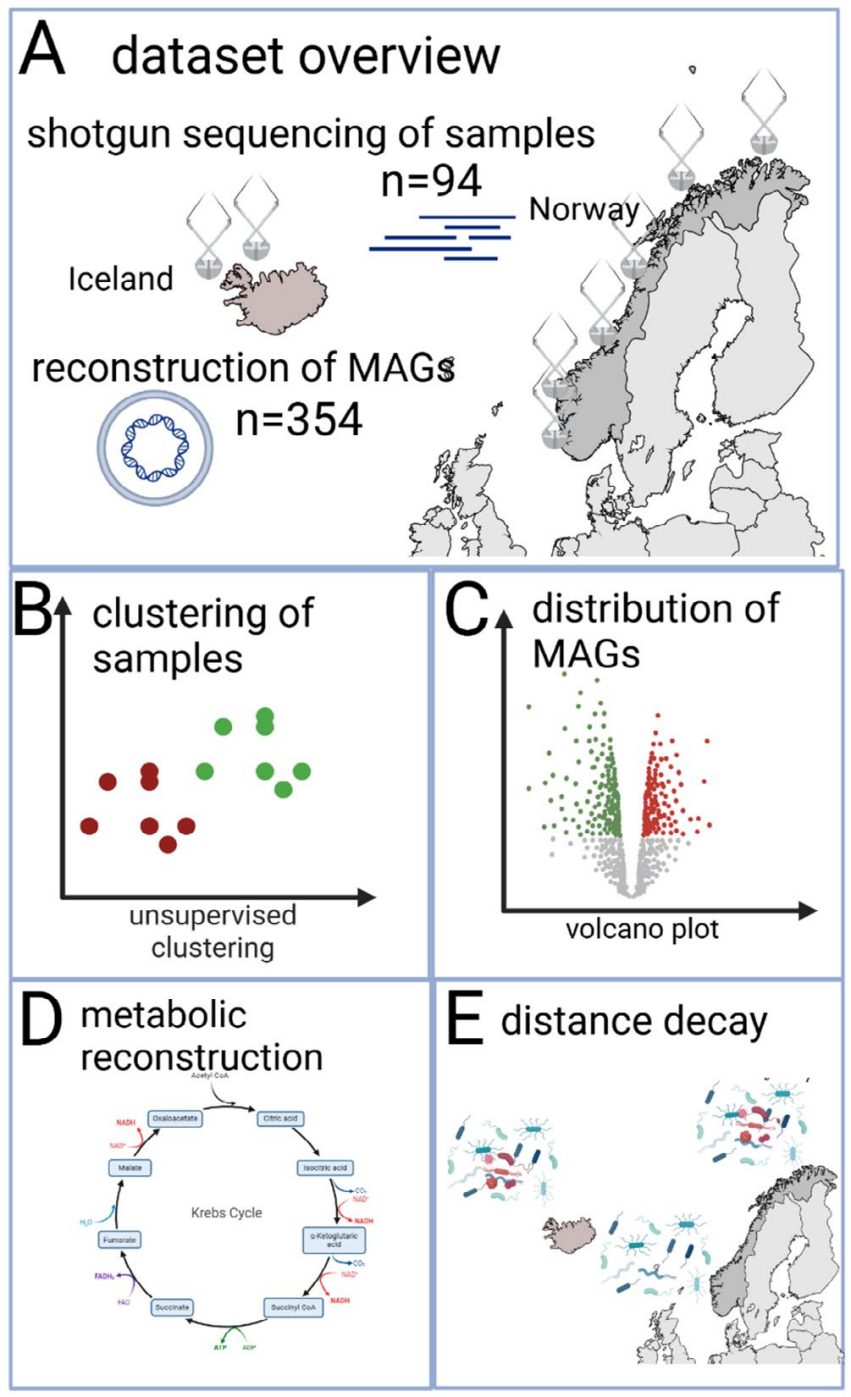
Analytical strategy used in this work. (A) Dataset overview showing sampling locations and the number of MAGs and shotgun sequences analysed. The dataset consisted of 94 samples, sampled along the Norwegian and the Icelandic coasts with a relative abundance of 356 high‐quality MAGs. (B) Clustering of samples using PCA to identify unsupervised clusters based on relative MAG abundance. (C) Volcano plots highlighting MAGs with significant differences between clusters. (D) Metabolic pathways and traits unique to each cluster identified through DRAM analyses. (E) Determination of the relationship between MAG abundance, functions and distance decay patterns.

An outline of the analytical strategy is presented in Figure 1.

## 2 Results

### 2.1 Taxonomic Diversity of MAGs

**FIGURE 2:**
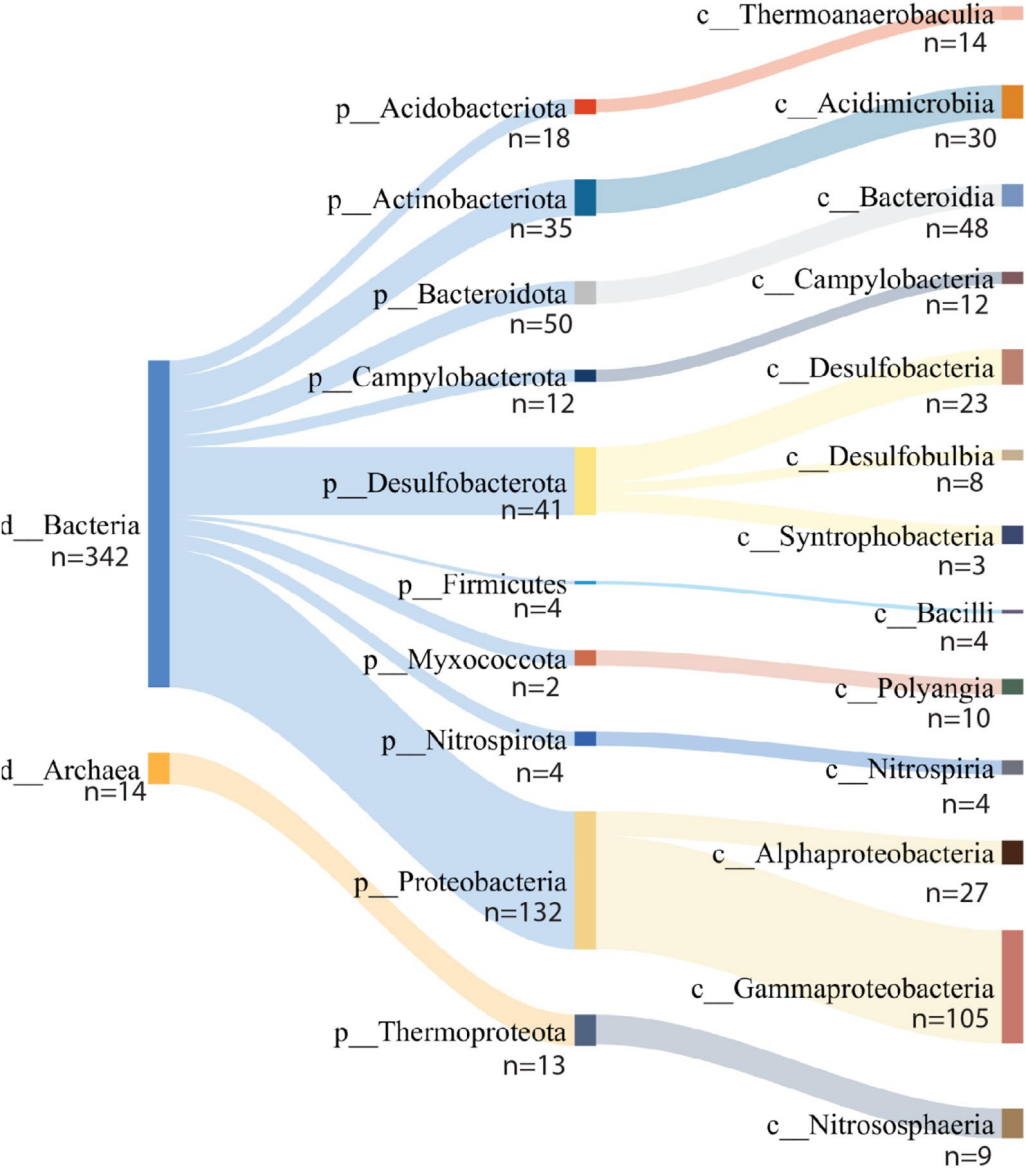
Overall taxonomic distribution of the MAGs. The relative distributions of the MAGs were determined at the domain to class level. The line widths illustrate the relative abundance, while the numbers illustrate the number of MAGs covered at the different taxonomic levels. The colour codes are included to highlight the associations.

To obtain an overview of the taxonomic composition of sediment microbial assemblages, we investigated the average relative distribution of MAGs across all samples. Figure 2 shows the taxonomic distribution of the MAGs from domain to class levels. For bacteria, Proteobacteria and Gammaproteobacteria exhibited both the highest relative abundance of metagenome sequence reads, as well as the largest number of MAGs. Archaea comprised a lower proportion of MAGs compared to Bacteria and were dominated by the phylum Thermoproteota and the class Nitrosophaeria.

### 2.2 Unsupervised Clustering Unveiled Distinct Patterns of MAG Relative Abundance Distribution

**FIGURE 3:**
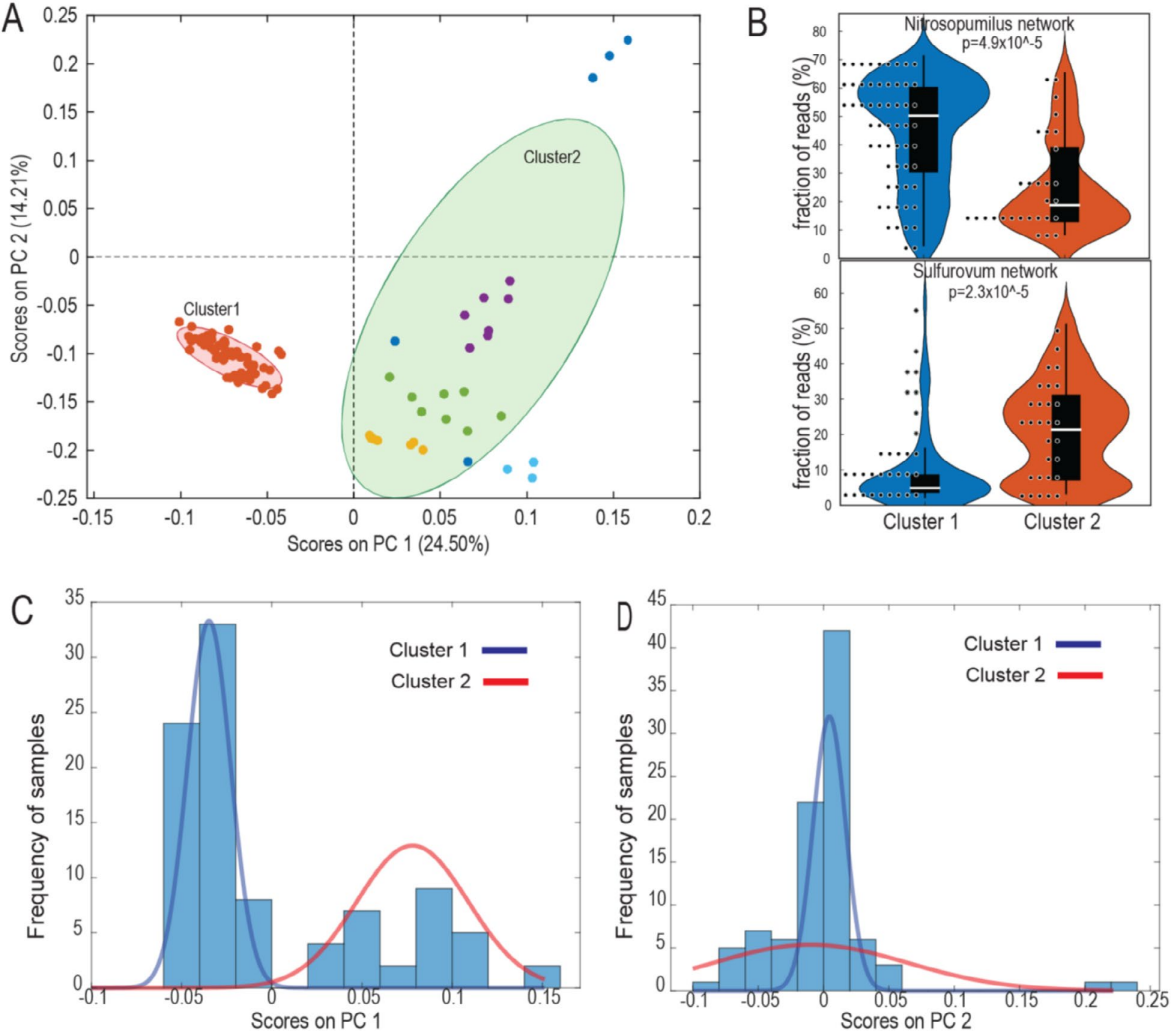
PCA analysis of the samples analysed based on the relative distribution of MAGs within each sample. (A) PCA plot illustrating the clustering pattern of samples. Dbscan identified five clusters, as visualised by the colours of the dots. The ellipses illustrate the 95% confidence interval for Clusters 1 and 2 after binarisation. (B) Illustration of the association between Clusters 1 and 2 samples as defined from the MAG distribution in the current work, and the fraction of 16S rRNA gene reads belonging to the Nitrosopumilus—and Sulfurovum networks, as determined from 16S rRNA gene sequence distribution for the same samples from the previous analyses (Nilsen et al. 2025). The *p* values were determined using the Kruskal–Wallis test. Histogram for the sample distribution along PC 1 (C) and PC 2 (D) with the respective fitted normal distributions.

We used PCA to perform an unsupervised investigation of intrinsic distribution patterns of the 354 MAGs across the 94 sampling sites investigated. This approach allows us to group samples that share similar MAG composition. We found a distinct distribution pattern of the samples, with one tight cluster associated with negative PCA scores, while the samples associated with positive scores showed a more scattered distribution (Figure 3A). To define clusters of samples within the dataset, we applied DBSCAN to handle datasets with varying cluster sizes and potential outliers. The DBSCAN analysis identified five clusters in the PCA data, with one dominant cluster encompassing 69% of the sampling sites. The remaining clusters were small, each accounting for less than 10% of the sampling sites, while three sites were identified as outliers. Due to the imbalance in cluster sizes, we binarised the data into two clusters as defined from the structuring along the first principal component (Figure 3C), since there was no clear clustering structure along the second component (Figure 3D). The large cluster (69% of the samples) was designated as Cluster 1, while the smaller clusters and outliers were grouped together as Cluster 2, representing 31% of the samples. A biplot with a 95% confidence interval confirmed no overlap between Clusters 1 and 2 (Figure 3A).

Based on 16S rRNA gene analyses, we have previously identified that the genera *Nitrosopumilus* and *Sulfurovum* centralise two main networks of North Atlantic seafloor microbiota. Since the samples included in our study have been 16S rRNA gene sequenced, we investigated how Clusters 1 and 2 were associated with the *Nitrosopumilus* and *Sulfurovum* networks, as determined from 16S rRNA gene relative abundance (Nilsen et al. 2024). The comparison unveiled a strong association of MAG Cluster 1 with the 16S rRNA gene *Nitrosopumilus* network and Cluster 2 with the *Sulfurovum* network (Figure 3B).

### 2.3 MAGs Show Distinct Distribution Between Clusters

**FIGURE 4:**
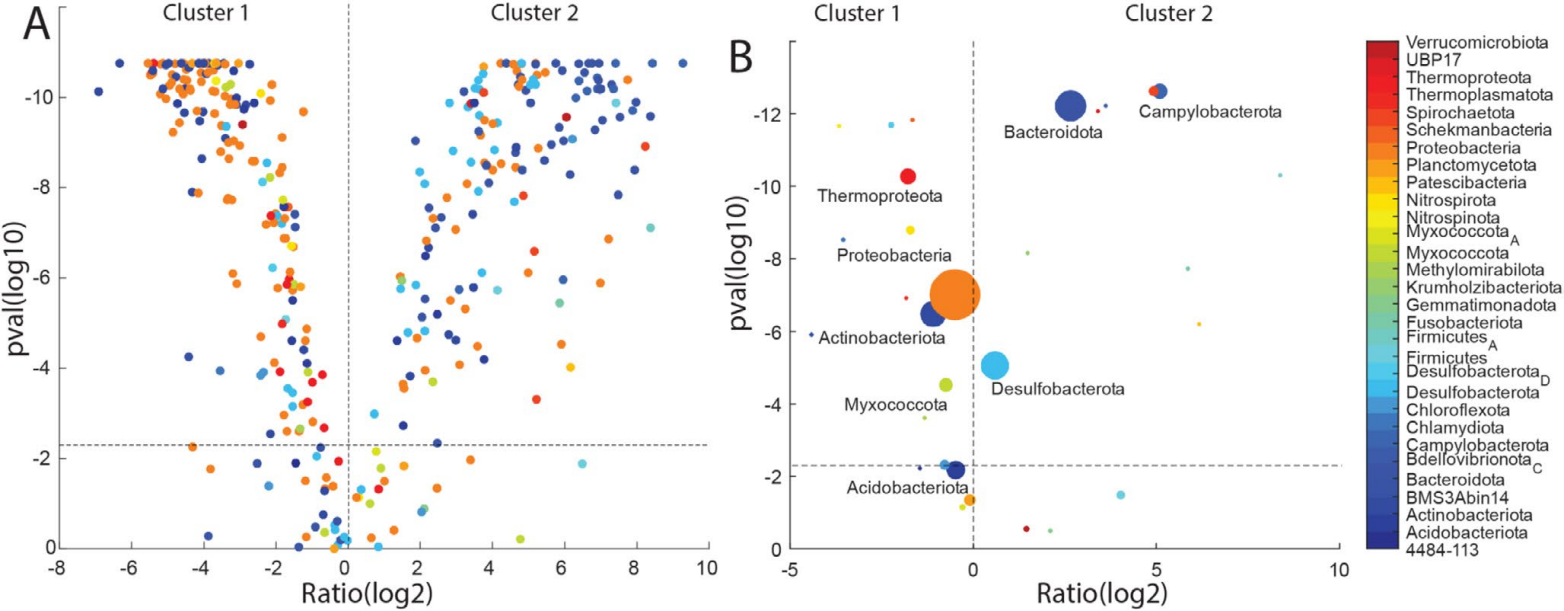
Distribution of MAGs in Cluster 1 and 2. The distribution pattern was determined using volcano plots. (A) Distribution of all the 356 high quality MAGs. (B) Distribution at the Phylum level. The size of the circles represents the number of MAGs within each phylum. The colour code represents the different phyla. For both panels the *p* values represent the FDR corrected values from the Kruskal–Wallis test. The log2 ratio represents the log2 of the ratio of the relative abundance of the respective MAGs/phyla in Cluster 2 divided by Cluster 1.

We identified 167 MAGs that met the criteria for being significantly associated with Cluster 1. These MAGs showed at least a 2‐fold higher abundance in samples from Cluster 1 compared to Cluster 2 and demonstrated statistically significant differences in abundance between the two clusters (*p* < 0.05, with False Discovery Rate (FDR) correction applied using the Kruskal–Wallis test). Similarly, 150 MAGs satisfied the same criteria for significant association with Cluster 2. Only 39 MAGs showed no statistically significant difference in abundance between the two clusters, indicating that most MAGs are distinctly associated with either Cluster 1 or Cluster 2. These associations between MAGs and their respective clusters are visually represented in Figure 4A.

The phylum Thermoproteota showed a statistically significant difference in differential abundance between the clusters, with the highest abundance in Cluster 1. In contrast, the phyla Campylobacteriota and Bacteroidota showed the highest differential abundance in Cluster 2. Although Proteobacteria was the most abundant phylum overall across both clusters, it showed only slightly higher differential abundance in Cluster 1 as compared to Cluster 2 (Figure 4B).

The association between differential abundance and contamination levels in the MAGs was analysed using Spearman correlation. We found no statistically significant correlation between contamination and differential abundance (rho = 0.06, *p* = 0.28).

### 2.4 Major Functional Differences Between Clusters

We used the functional modules identified by DRAM to determine the difference in genetic potential for MAGs associated with Clusters 1 and 2. Information about DRAM is given in Materials and Methods.

**FIGURE 5:**
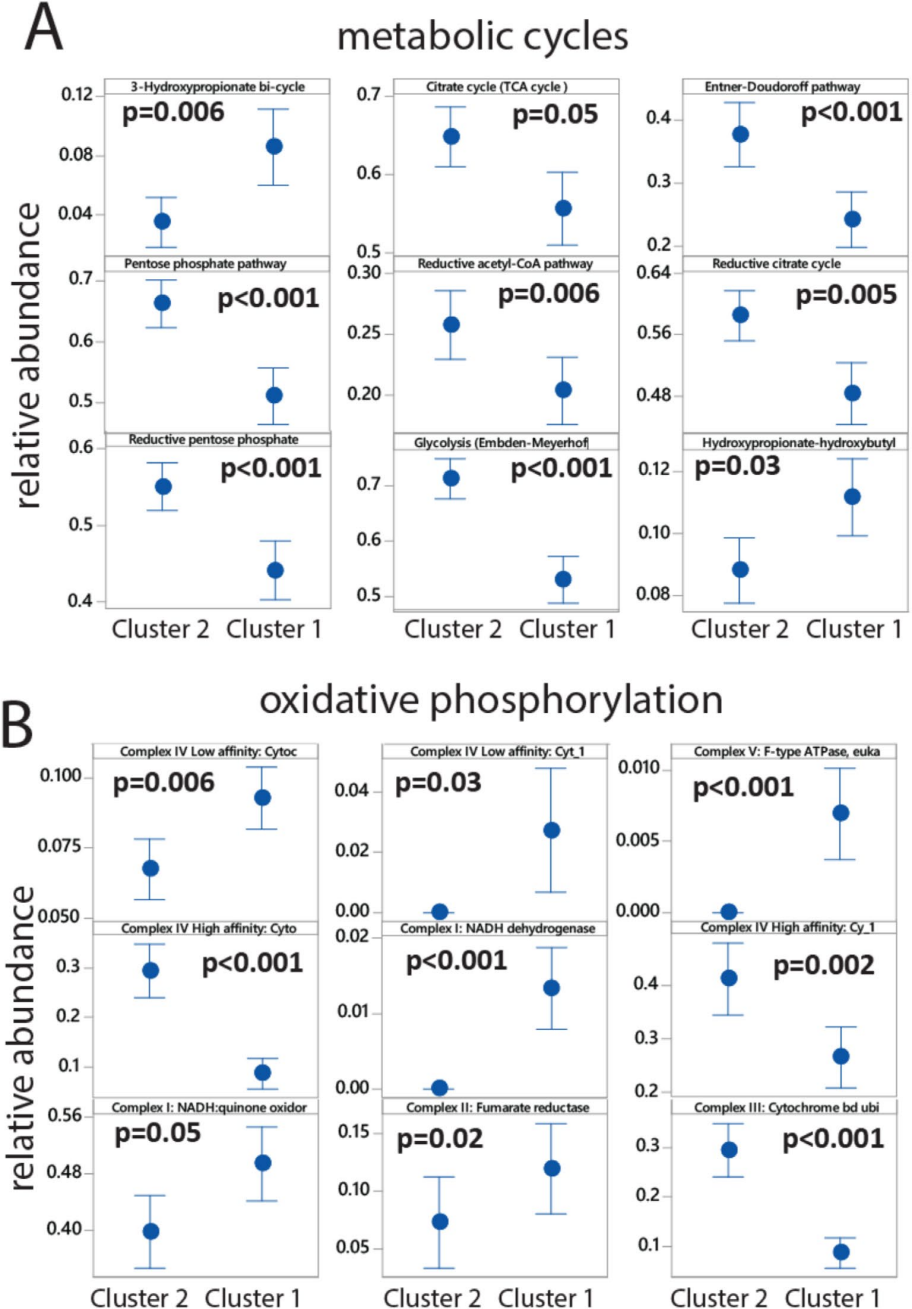
Association between the clusters and (A) metabolic cycles and (B) respiratory components. The components were extracted from the distilled output from DRAM. Only components showing a statistically significant (*p* < 0.05) after FDR correction for the Kruskal–Wallis test are shown.

The genetic potential for carbon dioxide fixation differed significantly between the two clusters. Cluster 1 showed an overrepresentation of the hydroxypropionate‐based cycles represented by 3‐hydroxypropionate bi‐cycle and hydroxypropionate‐hydroxybutyl cycle, while Cluster 2 was enriched in the reductive acetyl‐CoA pathway (Wood‐Ljungdahl pathway), the reductive citrate cycle (Arnon‐Buchanan cycle) and the reductive pentose phosphate cycle (Calvin cycle) for carbon fixation (Figure 5A).

For oxidative phosphorylation, Cluster 1 was characterised by an overrepresentation of low‐affinity cytochrome C, whereas Cluster 2 exhibited a higher abundance of high‐affinity cytochrome C (Figure 5B).

**FIGURE 6:**
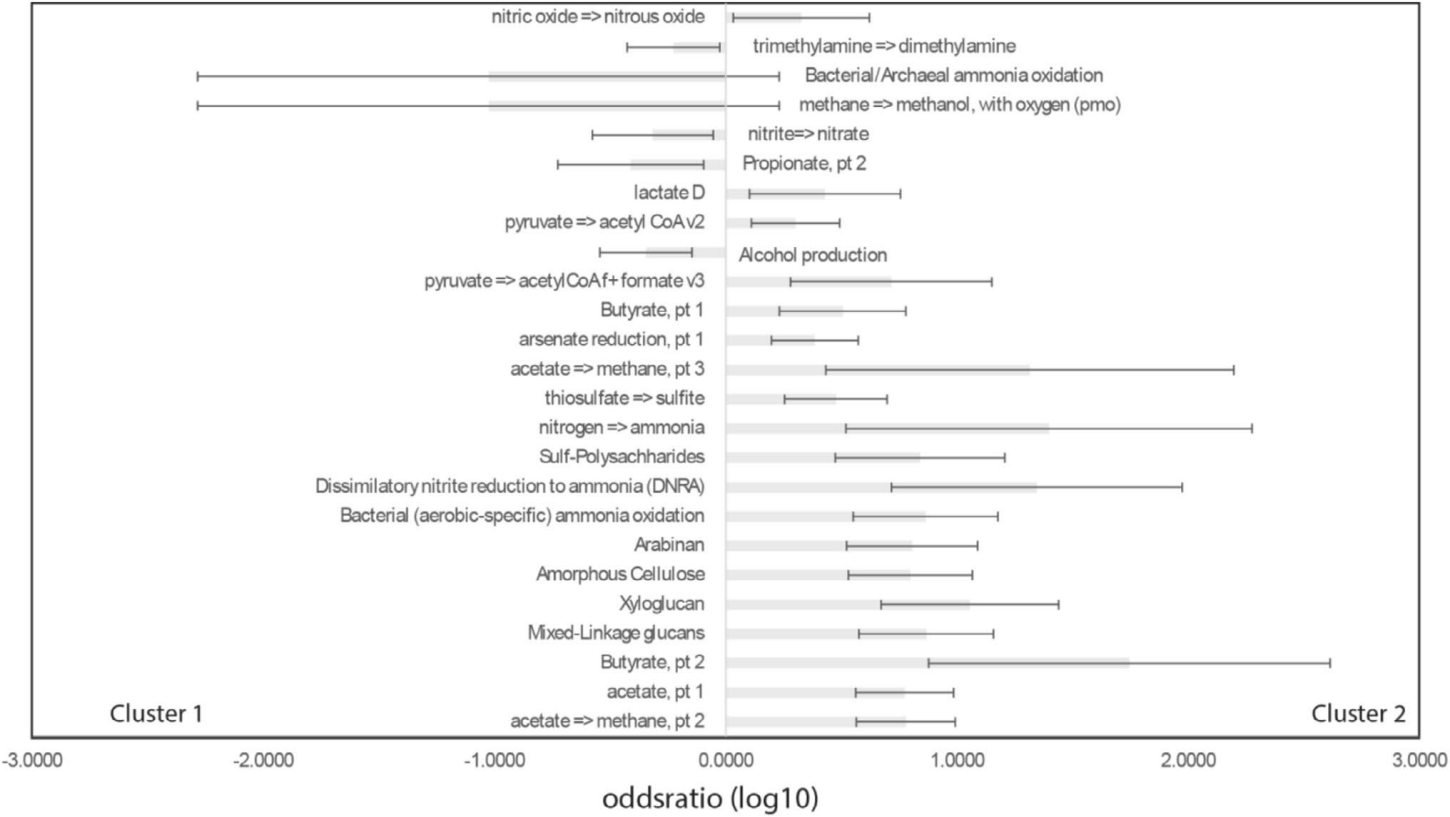
Functional associations of the identified clusters. The functional associations were determined based on the categorical output from the DRAM analyses. The error bars represent the 95% confidence interval for the odds ratios.

Cluster 2 had the highest number of overrepresented categorical traits, as derived from the DRAM analyses (Shaffer et al. 2020), with 19 distinct functions compared to only 6 in Cluster 1. Notably, Cluster 2 displayed enrichment in functions such as dissimilatory nitrate reduction to ammonium (DNRA) and nitrogen fixation. In contrast, Cluster 1 showed strong overrepresentation in pathways related to propionate metabolism, bacterial/archaeal ammonium oxidation and nitrite oxidation to nitrate (Figure 6 and S1).

### 2.5 Chemical, Physical and Physiochemical Associations of the Clusters

**FIGURE 7:**
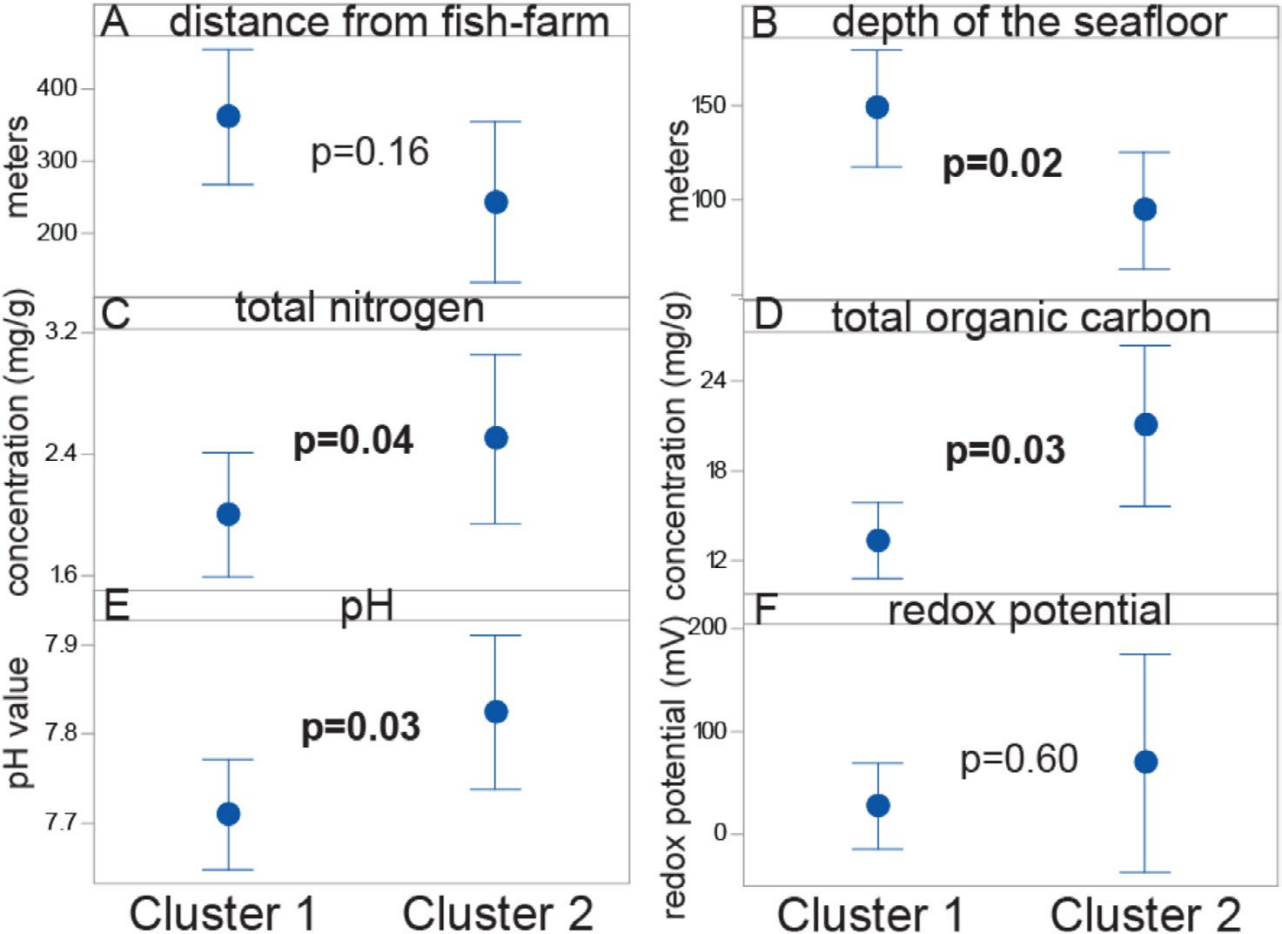
Physical, chemical and physiochemical associations of Cluster 1 and 2. We determined the physical (A and B), the chemical (B and C), and the physiochemical (D and E) associations of the seafloor towards Cluster 1 and 2. The statistical significance was determined using the Kruskal–Wallis test. Statistically significant *p* values are marked in bold.

In analysing the physical and physiochemical associations, we found no statistically significant relationship between the MAGs cluster‐membership and their distance away from aquaculture facilities. However, there was a noticeable trend indicating that Cluster 2 may be overrepresented near these facilities (*p* = 0.16, Kruskal–Wallis test). In contrast, Cluster 1 MAGs were significantly associated with samples taken at greater depths. Both nitrogen and organic carbon levels were significantly positively linked to Cluster 2, which was also associated with higher pH values. However, no statistically significant differences were observed in redox potential between the clusters (Figure 7).

### 2.6 Geographical Distribution of the Clusters

**FIGURE 8:**
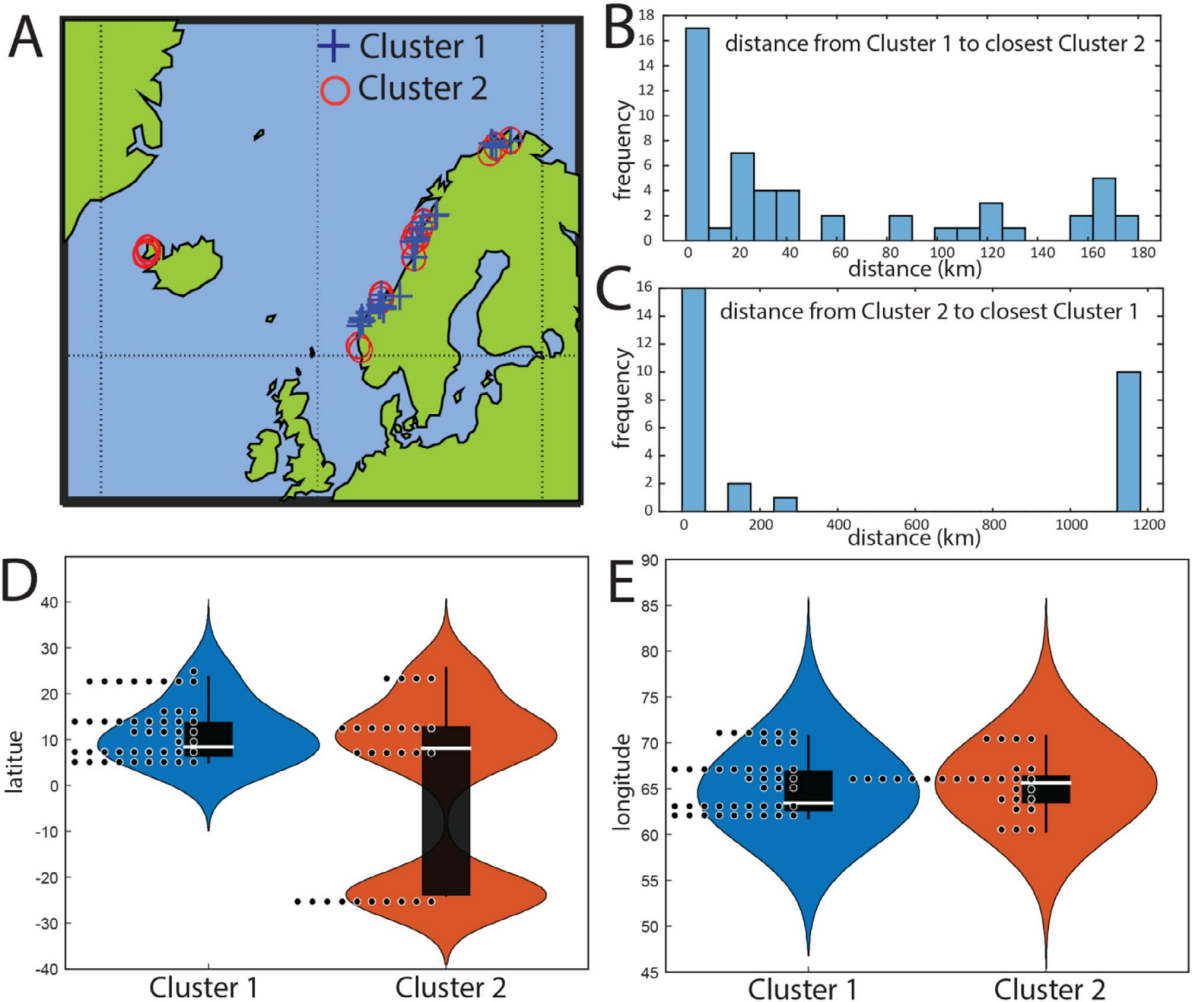
Geographical distribution of Clusters 1 and 2. (A) Geographic location of the clusters. (B and C) Distance from one cluster to the closest of the other clusters. (D and E) Latitudinal and longitudinal distribution of the clusters.

The geographic distribution of Clusters 1 and 2 sites along the Norwegian and Icelandic coasts is shown in Figure 8A. In most cases, Clusters 1 and 2 sites were located less than 10 km apart (Figure 8B,C). However, samples collected in Iceland exclusively belonged to Cluster 2, with the nearest Cluster 1 site located along the Norwegian coast, over 1000 km away (Figure 8C).

When examining latitude, Cluster 2 displayed a bimodal distribution, with sites present in both Iceland and Norway (Figure 8C). In contrast, longitude did not reveal any significant differences in the distribution of the two clusters (Figure 8D). This highlights the geographically dispersed nature of Cluster 2, whereas Cluster 1 was confined to the Norwegian coast.

### 2.7 Distance Decay Patterns of the MAGs

**FIGURE 9:**
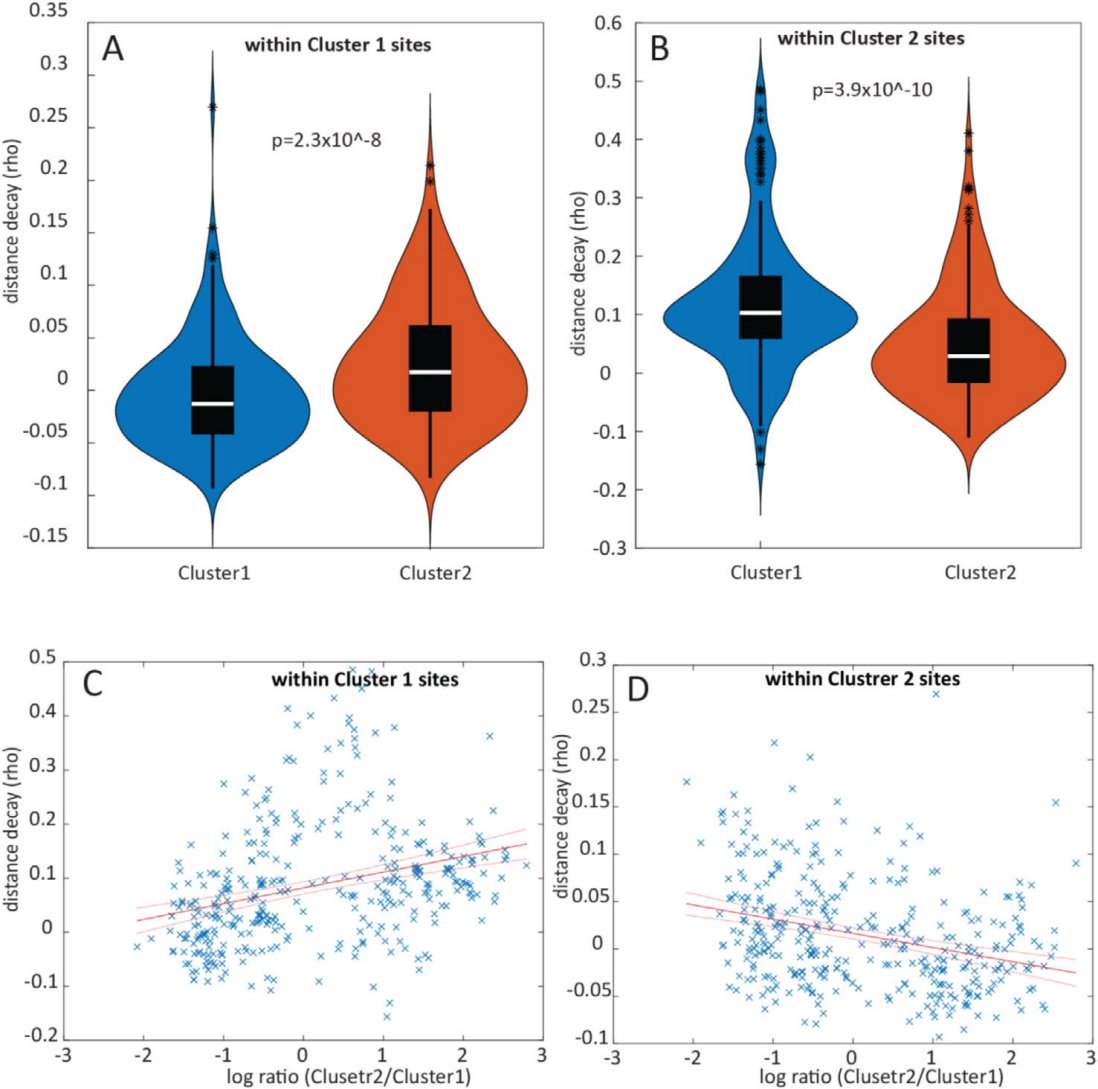
Distance decay patterns for MAGs. (A) Average distance decay for MAGs belonging to Clusters 1 and 2 within Cluster 1 sites, and (B) Cluster 2 sites. (C) Correlation between the log ratio (Cluster 2/Cluster 1) and distance decay for Cluster 1 sites and (D) Cluster 2 sites.

For each MAG the distance decay pattern was determined through the correlation between pairwise differences in the relative abundance for the MAG, and the pairwise geographical distances between all the 94 sites compared. In the analysis of MAGs associated with Clusters 1 and 2 sites, distinct patterns emerged based on their representation. MAGs that were proportionally abundant in these clusters exhibited little to no distance decay. Conversely, MAGs that were underrepresented in these clusters displayed a positive distance decay (Figure 9A,B).

We further investigated the association between the level of overrepresentation of the MAGs in the clusters and distance decay. This analysis revealed that the MAGs that showed the highest overrepresentation for the respective cluster also showed the lowest distance decay, while those that were underrepresented also showed the largest distance decay (Figure 9C,D).

## 3 Discussion

The key finding of our study is that different cosmopolitan MAGs played a central role in shaping clusters of microbes, irrespective of geographic distance. This suggests that microbial cluster formation in coastal ecosystems is primarily driven by niche selection, as has previously been observed for hydrothermal vents (Dick 2019).

Based on the correlation structure between distance decay and the Cluster 2 to 1 ratio for the MAGs, it appears that niche selection operates at the level of microbial networks rather than individual microorganisms. This interpretation is supported by the observation that microorganisms which tend to co‐occur do so regardless of whether the networks are geographic distance. Recent theoretical work suggests that microbial cooperation is thermodynamically favourable, offering a potential mechanistic basis for selection at the network level (Seto and Kondoh 2023). Furthermore, microorganisms that were not central to their respective clusters exhibited a more pronounced distance decay pattern. This indicates that when microorganisms are not subject to positive selection, their distribution resembles neutral assembly processes (McGill et al. 2006). Consequently, we propose a model for seafloor microbiota assembly that involves a combination of positive selection and neutral assembly processes. This could help resolve the long‐standing debate in marine microbiology about the observed microbial diversity being far greater than the number of ecological niches (Ward and Collins 2022).

Cluster 2 is predominantly associated with reductive and fermentative pathways such as nitric oxide to nitrous oxide conversion, lactate metabolism, DNRA and acetate to methane production, which are indicative of anaerobic or low‐oxygen environments (Brune et al. 2000; Ulloa et al. 2012). In contrast, Cluster 1 features pathways like trimethylamine to dimethylamine transformation, bacterial/archaeal ammonia oxidation, methane to methanol conversion and alcohol production, suggesting a predominance of oxidative and ammonia‐based metabolic processes, likely in oxygen‐rich settings (Edwards et al. 2005).

The chemical composition shows an enrichment of both nitrogen and organic carbon for Cluster 2. This supports the genetic findings of the association of Cluster 2 with organic enrichment. Surprisingly, however, Cluster 1 was associated with a lower pH in the sediment than Cluster 2. The lower pH for samples belonging to Cluster 1 could be related to the *Nitrosopumilus* association and nitrification process, which will lower pH (Hutchins and Capone 2022). The depth association could potentially be related to the samples being less affected by anthropogenic activity.

The tight clustering of Cluster 1, connected with the distribution along the whole Norwegian coast, and the connection to low organic carbon and nitrogen, support that this cluster could represent a common state for the undisturbed seafloor. This state is potentially driven by cobalamin‐dependent carbon dioxide fixation and ammonium oxidation to nitrate. Cluster 2 may therefore represent deteriorated ecological states caused by geothermal and anthropogenic impact. The more scattered distribution of this cluster could potentially be explained by the Anna Karenina principle that multiple states exist for microbiota deterioration while there are few functioning states (Zaneveld et al. 2017).

In conclusion, our findings suggest that microbial communities on the seafloor are centralised around key functions, with environmental selection driving the formation of these clusters across regions. The existence of a core microbiota further supports the idea that environmental factors, rather than geographical distance, shape microbial distributions at the coastal seafloor.

## 4 Materials and Methods

### 4.1 Dataset

We have utilised a subset of 94 shotgun sequenced samples from the 1546 samples for which 356 MAGs were retrieved by Nilsen et al. (Nilsen et al. 2025). Van Veen grab samples (0.1 m^2^ each) were taken following the guidelines of Norwegian standard NS 9410. Chemical parameters and eDNA were analysed using material from the upper 1 cm of sediment, while total organic carbon (TOC) was assessed from the top 5 cm. A summary of the samples included is given in Table S1.

Sequencing was done with an average sequencing depth of 133 million paired‐end reads per sample. Briefly, the whole genome sequencing reads were trimmed, filtered and merged using Bbmap v39.01. Assemblies were generated with SPAdes v3.15.5 (Nurk et al. 2017). Contig coverages were calculated with CoverM v0.6.1, and bins were created using MaxBin2 v2.2.7 (Wu et al. 2015) and MetaBat2 v2.15 (Kang et al. 2015). Bins were evaluated with checkM2 v1.0.1 (Chklovski et al. 2022) and de‐replicated using dRep v3.4.0 (Olm et al. 2017). MAGs with ≥ 75% completeness and < 25% contamination were taxonomically classified with GTDB v2.3.2 (Chaumeil et al. 2019), and functionally annotated with DRAM v1.4 (Shaffer et al. 2020). The inclusion of MAGs with relatively high contamination levels is due to the challenges associated with assembling seafloor metagenomic samples.

The functional analyses were done using distilled and refined annotation of metabolism (DRAM), which is a bioinformatics pipeline for automated annotation of MAGs into a catalogue of traits (Shaffer et al. 2020). The traits used in our study include 13 metabolic pathways, five electron transport complexes, 19 pathways for polysaccharide degradation, 12 modules for nitrogen metabolism, four modules for sulphur metabolism, 11 modules for methanogenesis and methanotrophy, 13 modules for short‐chain fatty acid and alcohol conversion, in addition to five other reductases. The metabolic pathways and electron transport complexes are presented by the fraction of the fraction covered, while modules are represented by presence/absence.

### 4.2 Data Analysis

All data analyses were done in the MATLAB programming environment (MathWorks, California). We performed principal component analysis (PCA) using the samples as objects and the relative abundance of the MAGs as features. The PCA ordination reflects the similarity of the samples with respect to the relative distribution of the MAGs within each sample. Density‐based spatial clustering of applications with noise (DBSCAN) was used for determining the number of clusters for PCA compressed data, with the minimum number of two members for being considered a class. The largest distance for samples considered to be related was automatically determined by the range and number of datapoints. PCA and DBSCAN were performed using the MATLAB implementation of PLS Toolbox (Eigenvector, Washington).

For the distance decay analyses we made two vectors for each MAG. The first vector contained the absolute value of all the pairwise differences in relative abundance across all 94 sites included. The second vector contained the corresponding geographical distances between the sites. Then the correlation between these vectors was determined by Spearman correlation. A positive correlation between geographical distance and difference in relative abundance is considered as distance decay, meaning that microbial communities become increasingly different in their composition as the geographic distance between them grows.

For the statistical analyses, we used Spearman correlations as implemented in the Matlab corr function if two continuous variables were compared. If one continuous and one categorical variable were compared, we used the Kruskal–Wallis test, as implemented in the Kruskal–Wallis function. If two categorical variables were compared, we used the Matlab crosstab function. Odds ratios were calculated for the categorical data. For the odds ratios, the 95% confidence intervals were calculated as described by Altman (Altman 1991). False discovery rate (fdr) was corrected using the Benjamini–Hochberg method implemented in the mafdr function.

### 4.3 Plotting and Visualisation

Interval plots were made in MINITAB (Minitab, Pennsylvania), while the rest of the plots were made in Matlab. The violin plots were generated using the daviviolin module(github.com/povilaskarvelis/DataViz). The Sankey diagram was made using the SSsankey module (/www.mathworks.com/matlabcentral/fileexchange/128679‐sankey‐plot). The other plots were made using standard plotting methods implemented in Matlab.

## Author Contributions

K.R.: Wrote the paper and analyzed data. T.N.: Conducted initial analyses and commented on paper. R.P.: Responsible for sampling and commented on paper. N.B.K.: Provided scientific input and commented on paper. J.L.R.: Provided scientific input and commented on paper. S.M.: Provided scientific input and commented on paper. M.S.: Responsible for sampling and commented on paper. A.H.: Responsible for sampling and commented on paper. I.L.A.: Conducted initial analyses and commented on paper. M.P.: Conducted initial analyses and commented on paper. J.M.: Conducted initial analyses and commented on paper. M.Ø.S.: Provided scientific input and commented on paper. L.G.S.: Analyzed the data and commented on paper.

## Conflicts of Interest

The authors declare no conflicts of interest.

## Supporting information


**Data S1.** Supporting Information.

## Data Availability

The data that support the findings of this study are openly available in SRA at https://www.ncbi.nlm.nih.gov/, reference number PRJNA1128851.
