# Supplementary material for: The Coastal Seafloor Microbiota Is Structured by Local Selection of Cosmopolitan Taxa
Source: Environ Microbiol Rep. 2025 Jun 19;17(3):e70123. doi: 10.1111/1758-2229.70123 (PMC12178751; doi:10.1111/1758-2229.70123)
Supplement: Supplementary file 1 — Data S1. Supporting Information. [file EMI4-17-e70123-s001.docx]

**SUPPLEMENTARY FIGURES**


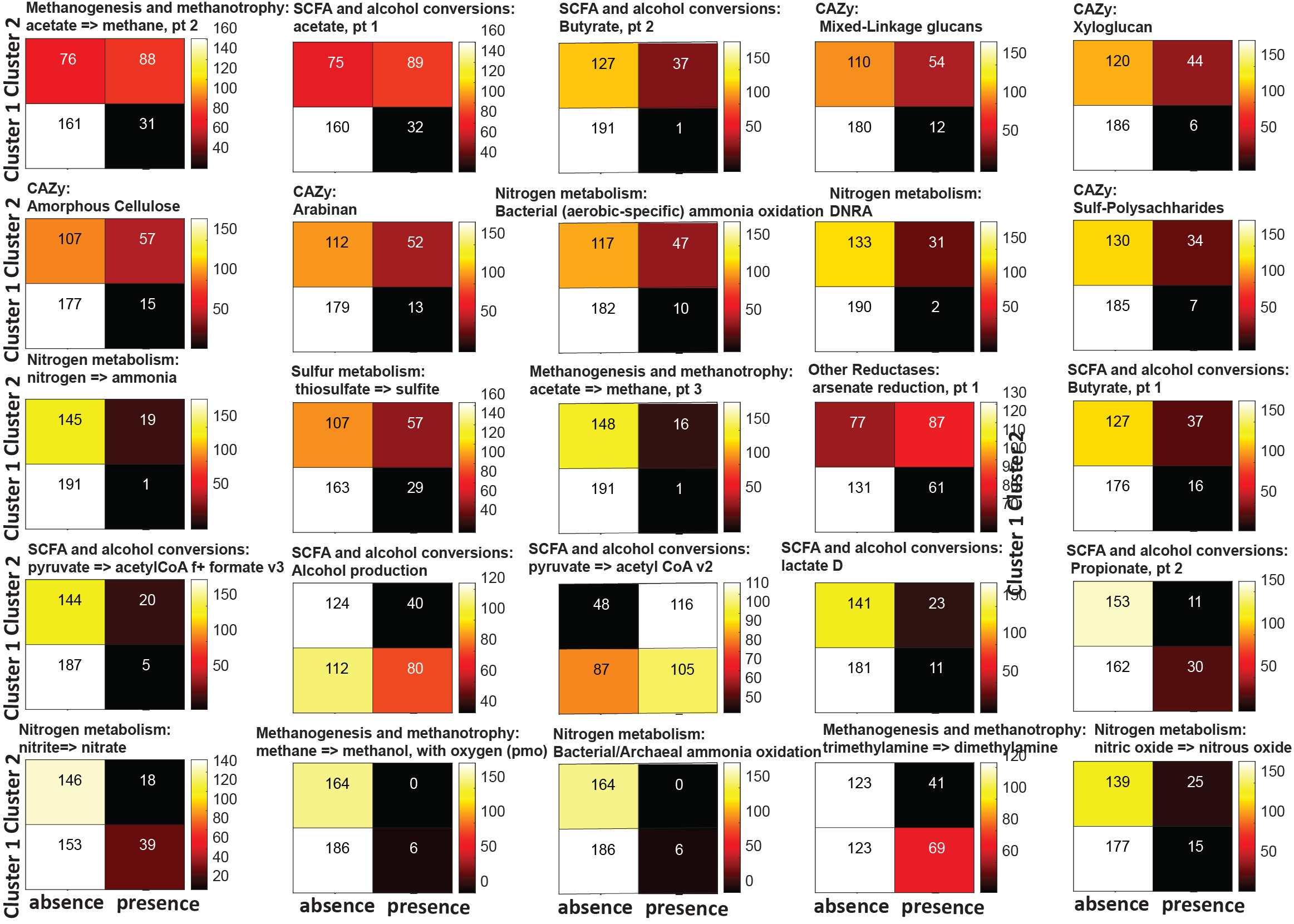


**Suppl. Figure 1 Distribution of categorical functions from the distilled DRAM output.** Only functions with a FDR corrected p-value < 0.05 is shown. The statistical testing was done using the Chi Square test.

**SUPPLEMENTARY TABLES**

**Suppl. Table 1. Metadata for the shotgun sequenced samples**

| **Sample#^1^** | **Country** | **County** | **Ecological state^2^** | **Depth** | **Farm-distance** | **TN^3^** | **TOC^4^** | **pH** | **Eobs^5^** |
| --- | --- | --- | --- | --- | --- | --- | --- | --- | --- |
| 37 | Norway | Nordland |  | 150 | 1107 |  |  |  | 425 |
| 82 | Norway | Nordland |  |  |  |  |  |  |  |
| 85 | Norway | Nordland |  |  |  |  |  |  |  |
| 22 | Norway | Nordland | Bad (0.2-0.4) | 123 | 0 | 3.6 | 22.9 | 7.9 | -6 |
| 111 | Norway | Nordland | Very good (0.8-1) | 182 | 3760 | 0.6 | 3.22 | 8.03 | 111.3 |
| 124 | Norway | Nordland | Very good (0.8-1) | 182 | 3760 | 0.6 | 3.22 | 8.03 | 111.3 |
| 163 | Norway | Nordland |  | 50 | 912 |  |  |  | 364 |
| 176 | Norway | Nordland |  | 50 | 912 |  |  |  | 364 |
| 121 | Norway | Nordland | Very good (0.8-1) | 51 | 0 | 0.9 | 4 | 8.21 | 160 |
| 218 | Norway | Nordland | Very good (0.8-1) | 215 | 1065 | 1.7 | 13.6 | 7.86 | 58 |
| 219 | Norway | Nordland | Very good (0.8-1) | 215 | 1065 | 1.7 | 13.6 | 7.86 | 58 |
| 277 | Norway | Nordland | Good (0.6-0.8) | 84 | 1349 | 1.1 | 7.8 | 7.97 | 31 |
| 351 | Norway | Nordland | Very good (0.8-1) | 197 | 4900 | 2.3 | 14.8 | 7.57 | 139.5 |
| 358 | Norway | Nordland | Very good (0.8-1) | 197 | 4900 | 2.3 | 14.8 | 7.57 | 139.5 |
| 490 | Norway | Troms_Finnmark | Very good (0.8-1) | 78 | 1158 | 1.7 | 7.7 |  |  |
| 472 | Norway | Troms_Finnmark | Very good (0.8-1) | 101 | 25 | 1.9 | 8.5 | 7.4 |  |
| 522 | Norway | Troms_Finnmark | Very good (0.8-1) | 192 | 25 | 1.7 | 7.6 | 7 |  |
| 481 | Norway | Troms_Finnmark | Very good (0.8-1) | 78 | 1158 | 1.7 | 7.7 |  |  |
| 676 | Norway | Troms_Finnmark |  |  |  |  |  |  |  |
| 709 | Norway | Troms_Finnmark | Very good (0.8-1) |  |  | 1.6 | 9.6 | 7.6 |  |
| 752 | Norway | Troms_Finnmark | Very good (0.8-1) |  |  | 0.9 | 5.2 | 7.9 |  |
| 850 | Norway | Møre_Romsdal | Very good (0.8-1) | 277 | 1100 | 3.7 |  | 7.47 | -106 |
| 853 | Norway | Møre_Romsdal | Very good (0.8-1) | 277 | 1100 | 3.7 |  | 7.47 | -106 |
| 897 | Norway | Møre_Romsdal | Very good (0.8-1) | 87 | 30 | 1.6 | 14.7 | 7.76 | 48.8 |
| 923 | Norway | Møre_Romsdal | Very good (0.8-1) | 143 | 1000 | 2.9 | 17.8 | 7.72 | -24 |
| 924 | Norway | Møre_Romsdal | Very good (0.8-1) | 143 | 1000 | 2.9 | 17.8 | 7.72 | -24 |
| 958 | Norway | Møre_Romsdal | Very good (0.8-1) | 61 | 1247 | 2.9 | 19.1 | 7.7 | -20 |
| 963 | Norway | Møre_Romsdal | Very good (0.8-1) | 61 | 1247 | 2.9 | 19.1 | 7.7 | -20 |
| 985 | Norway | Møre_Romsdal | Very good (0.8-1) | 72 | 1113 | 1.7 | 10.7 | 7.69 | 149 |
| 986 | Norway | Møre_Romsdal | Very good (0.8-1) | 72 | 1113 | 1.7 | 10.7 | 7.69 | 149 |
| 918 | Norway | Møre_Romsdal | Very good (0.8-1) | 119 | 25 | 1.6 | 13.2 | 7.68 | -87 |
| 951 | Norway | Møre_Romsdal | Very good (0.8-1) | 53 | 25 | 2.1 | 17.7 | 7.66 | 41 |
| 981 | Norway | Møre_Romsdal | Very good (0.8-1) | 74 | 30 | 2.5 | 16 | 7.45 | -3 |
| 1003 | Norway | Møre_Romsdal | Moderate (0.4-0.6) | 85 | 926 |  | 33.4 | 7.86 | 197 |
| 1004 | Norway | Møre_Romsdal | Moderate (0.4-0.6) | 85 | 926 |  | 33.4 | 7.86 | 197 |
| 1084 | Norway | Møre_Romsdal | Very good (0.8-1) | 124 | 1348 | 2.2 | 15.2 | 7.61 | 45.1 |
| 1027 | Norway | Møre_Romsdal | Very good (0.8-1) | 91 | 26 | 1.7 | 17.7 | 7.48 | -34 |
| 1053 | Norway | Møre_Romsdal | Very good (0.8-1) | 148 | 1128 | 0.7 |  | 7.43 | -137.1 |
| 1077 | Norway | Møre_Romsdal | Very good (0.8-1) | 101 | 28 | 1.1 | 12.5 | 7.57 | 165 |
| 1105 | Norway | Møre_Romsdal | Very good (0.8-1) | 272 | 1058 | 2.5 |  | 8.06 | -49 |
| 1138 | Norway | Møre_Romsdal | Very good (0.8-1) | 106 | 1430 | 1.5 | 17.9 | 7.63 |  |
| 1171 | Norway | Møre_Romsdal | Very good (0.8-1) | 154 | 1200 | 3.1 | 19.5 | 7.68 | 63 |
| 1176 | Norway | Møre_Romsdal | Very good (0.8-1) | 154 | 1200 | 3.1 | 19.5 | 7.68 | 63 |
| 1131 | Norway | Møre_Romsdal | Very good (0.8-1) | 119 | 25 | 2.8 | 26.9 | 7.73 |  |
| 1214 | Norway | Møre_Romsdal | Very good (0.8-1) | 91 | 1230 | 1.2 | 7.8 | 7.65 | 16 |
| 1217 | Norway | Møre_Romsdal | Very good (0.8-1) | 91 | 1230 | 1.2 | 7.8 | 7.65 | 16 |
| 1204 | Norway | Møre_Romsdal | Very good (0.8-1) | 70 | 28 | 0.9 | 6.3 | 7.75 | -66 |
| 1271 | Norway | Vestland | Very good (0.8-1) | 199 | 29 | 1.8 | 24.7 | 7.55 |  |
| 1496 | Iceland | Dýrafjörður | Good (0.6-0.8) |  |  | 2.6 | 24.3 | 7.81 |  |
| 136 | Norway | Nordland | Very bad (0-0.2) | 52 | 30 | 2.4 | 25.3 | 7.66 | 38 |
| 52 | Norway | Nordland |  | 90 | 212 |  |  |  | 90.3 |
| 96 | Norway | Nordland |  |  |  |  |  |  |  |
| 248 | Norway | Nordland | Very good (0.8-1) | 97 | 1350 | 2.1 | 12.8 | 7.76 | 0.52 |
| 206 | Norway | Nordland |  | 91 | 0 |  |  |  | -55 |
| 284 | Norway | Nordland | Very good (0.8-1) | 117 | 0 | 3.7 | 20.4 | 7.78 | -86 |
| 399 | Norway | Nordland |  | 135 | 1220 |  |  |  | -27.6 |
| 413 | Norway | Nordland |  | 135 | 1220 |  |  |  | -27.6 |
| 449 | Norway | Troms_Finnmark | Very good (0.8-1) | 148 | 1960 | 1.7 | 7.9 |  |  |
| 450 | Norway | Troms_Finnmark | Very good (0.8-1) | 148 | 1960 | 1.7 | 7.9 |  |  |
| 384 | Norway | Nordland | Very good (0.8-1) | 210 | 0 | 1.3 | 9.8 | 7.65 | -110 |
| 411 | Norway | Nordland |  | 139 | 0 |  |  |  | 6.5 |
| 444 | Norway | Nordland | Very good (0.8-1) | 59 | 6 | 1 | 6.1 | 7.88 | -21 |
| 463 | Norway | Troms_Finnmark | Good (0.6-0.8) | 66 | 25 | 1.4 | 7 | 7.5 |  |
| 507 | Norway | Troms_Finnmark | Bad (0.2-0.4) | 73 | 15 | 2.5 | 15 | 7.4 |  |
| 551 | Norway | Troms_Finnmark | Very bad (0-0.2) | 94 | 20 | 1.9 | 14 | 7.3 |  |
| 575 | Norway | Troms_Finnmark | Very bad (0-0.2) | 69 | 15 | 1.2 | 11 | 7.5 |  |
| 664 | Norway | Troms_Finnmark | Good (0.6-0.8) | 68 | 20 | 1.4 | 9.4 | 7.8 |  |
| 683 | Norway | Troms_Finnmark | Very bad (0-0.2) | 73 | 7 | 3 | 67 | 6.9 |  |
| 754 | Norway | Troms_Finnmark | Very good (0.8-1) |  |  | 0.9 | 5.2 | 7.9 |  |
| 832 | Norway | Møre_Romsdal | Very good (0.8-1) | 164 | 1115 | 4.6 |  | 7.43 | -104.6 |
| 835 | Norway | Møre_Romsdal | Very good (0.8-1) | 164 | 1115 | 4.6 |  | 7.43 | -104.6 |
| 825 | Norway | Møre_Romsdal | Very good (0.8-1) | 175 | 23 | 4.3 |  | 7.46 | -81 |
| 869 | Norway | Møre_Romsdal | Good (0.6-0.8) | 294 | 30 | 4.2 |  | 7.69 | -159 |
| 1049 | Norway | Møre_Romsdal | Very good (0.8-1) | 148 | 1128 | 0.7 |  | 7.43 | -137.1 |
| 1058 | Norway | Møre_Romsdal | Very good (0.8-1) | 105 | 30 | 0.7 |  | 7.43 | -74.1 |
| 1102 | Norway | Møre_Romsdal | Very good (0.8-1) | 272 | 1058 | 2.5 |  | 8.06 | -49 |
| 1115 | Norway | Møre_Romsdal | Very good (0.8-1) | 270 | 30 | 2.2 |  | 7.42 | -109 |
| 1262 | Norway | Vestland | Good (0.6-0.8) | 260 | 1400 | 1.2 | 9.76 | 7.5 |  |
| 1264 | Norway | Vestland | Good (0.6-0.8) | 260 | 1400 | 1.2 | 9.76 | 7.5 |  |
| 1185 | Norway | Møre_Romsdal | Bad (0.2-0.4) | 178 | 28 | 8.6 | 51.9 | 7.66 | 23 |
| 1229 | Norway | Vestland | Moderate (0.4-0.6) | 6 |  | 0.0014 | 11.8 | 7.6 |  |
| 1234 | Norway | Vestland | Good (0.6-0.8) | 8 |  | 0.0006 | 5.96 | 7.7 |  |
| 1275 | Iceland | Dýrafjörður | Good (0.6-0.8) | 30 | 25 | 4.2 | 20.4 | 7.65 |  |
| 1295 | Iceland | Dýrafjörður | Good (0.6-0.8) | 30 | 25 | 4.2 | 20.4 | 7.65 |  |
| 1320 | Iceland | Patreksfjörður | Good (0.6-0.8) | 54 | 25 | 3 | 45.2 | 7.88 |  |
| 1368 | Iceland | Dyrafjörður | Moderate (0.4-0.6) | 34 | 25 | 3.7 | 18.2 | 7.6 |  |
| 1388 | Iceland | Dyrafjörður | Moderate (0.4-0.6) | 34 | 25 | 3.7 | 18.2 | 7.6 |  |
| 1406 | Iceland | Tálknafjörður | Bad (0.2-0.4) | 58 | 25 | 3.7 | 33.9 | 7.87 |  |
| 1425 | Iceland | Tálknafjörður | Bad (0.2-0.4) | 51 | 35 | 3.1 | 39 | 7.8 |  |
| 1487 | Iceland | Dýrafjörður | Good (0.6-0.8) |  |  | 2.6 | 24.3 | 7.81 |  |
| 1498 | Iceland | Dýrafjörður | Good (0.6-0.8) | 44 | 25 | 2.9 | 21.7 | 7.69 |  |
| 1509 | Iceland | Dýrafjörður | Good (0.6-0.8) | 44 | 25 | 2.9 | 21.7 | 7.69 |  |
| 1517 | Iceland | Patreksfjörður | Good (0.6-0.8) | 57 | 500 | 2.1 | 41.9 | 7.9 |  |
| 1567 | Iceland | Dýrafjörður | Good (0.6-0.8) | 42 | 25 | 4 | 25 | 7.7 |  |

**^1^** numbering is according to the sample number in Nilsen et al. 2025 ^2^The ecological sate was determined using the nEQR index ^3^Total nitrogen in mg/kg, ^4^Total organic carbon in mg/kg, ^5^Redox potential in mVolt
